# Association of Naloxone Coprescription Laws With Naloxone Prescription Dispensing in the United States

**DOI:** 10.1001/jamanetworkopen.2019.6215

**Published:** 2019-06-21

**Authors:** Minji Sohn, Jeffery C. Talbert, Zhengyan Huang, Michelle R. Lofwall, Patricia R. Freeman

**Affiliations:** 1 College of Pharmacy, Ferris State University, Big Rapids, Michigan; 2 Institute for Pharmaceutical Outcomes and Policy, College of Pharmacy, University of Kentucky, Lexington; 3 Department of Biostatistics, College of Public Health, University of Kentucky, Lexington; 4 Center on Drug and Alcohol Research, University of Kentucky, Lexington

## Abstract

**Importance:**

To mitigate the opioid overdose crisis, states have implemented a variety of legal interventions aimed at increasing access to the opioid antagonist naloxone. Recently, Virginia and Vermont mandated the coprescription of naloxone for potentially at-risk patients.

**Objective:**

To assess the association between naloxone coprescription legal mandates and naloxone dispensing in retail pharmacies.

**Design, Setting, and Participants:**

This was a population-based, state-level cohort study. The sample included all prescriptions dispensed for naloxone in the retail pharmacy setting contained in IQVIA’s national prescription audit, which represents 90% of all retail pharmacies in the United States. The unit of observation was state-month and the study period was January 1, 2011, to December 31, 2017.

**Exposures:**

State legal intervention mandating naloxone coprescription.

**Main Outcomes and Measures:**

Number of naloxone prescriptions dispensed. State rates of naloxone prescriptions dispensed per month per 100 000 standard population were calculated.

**Results:**

The rate of naloxone dispensing increased after implementation of legal mandates for naloxone coprescription. An estimated 88 naloxone prescriptions per 100 000 were dispensed in Virginia and 111 prescriptions per 100 000 were dispensed in Vermont during the first full month the legal requirement was effective. In comparison, 16 naloxone prescriptions per 100 000 were dispensed in the 10 states (including the District of Columbia) with the highest opioid overdose death rates and 6 prescriptions per 100 000 were dispensed in the 39 remaining states. The number of naloxone prescriptions dispensed was associated with the legal mandate for naloxone coprescription (incidence rate ratio [IRR], 7.75; 95% CI, 1.22-49.35). Implementation of the naloxone coprescription mandate was associated with an estimated 214 additional naloxone prescriptions dispensed per month in the period following the mandates, holding all other variables constant. Among covariates, naloxone access laws (IRR, 1.37; 1.05-1.78), opioid overdose death rates (IRR, 1.06; 95% CI, 1.04-1.08), the percentage of naloxone prescriptions paid by third-party payers (IRR 1.009; 1.008-1.010), and time (IRR, 1.06; 95% CI, 1.05-1.07) were significantly associated with naloxone prescription dispensing.

**Conclusions and Relevance:**

These study findings suggest that legally mandated naloxone prescription for those at risk for opioid overdose may be associated with substantial increases in naloxone dispensing and further reduction in opioid-related harm.

## Key Points

**Question:**

Are legal mandates for naloxone coprescription associated with increases in naloxone prescription dispensing?

**Findings:**

In this population-based, state-level cohort study using data from retail pharmacies in all 50 states and the District of Columbia, having a legal mandate for naloxone coprescription was associated with approximately 7.75 times more dispensed naloxone prescriptions compared with not having the requirements. This equates to an estimated 214 additional naloxone prescriptions dispensed per month in the period following the mandates, holding all other variables constant.

**Meaning:**

State legal interventions that mandate naloxone coprescription for potentially at-risk patients may be associated with increases in naloxone prescription dispensing in retail pharmacies, and this strategy may be useful to improve naloxone availability and reduce opioid-related harm.

## Teaser

This population-based cohort study examines the rates of naloxone coprescription since implementation of state laws mandating coprescription for persons at risk for opioid overdose.

## Introduction

Deaths due to opioid overdose (OOD) have been continuously increasing over the past 2 decades, and this trend is predicted to continue.^1,2^ From 2016 to 2017, the age-adjusted rate of drug overdose deaths increased by 9.6%, from 19.8 per 100 000 standard population in 2016 to 21.7 per 100 000 in 2017.^2^ Of all drug overdose deaths, approximately 68% were attributed to opioids in 2017.^2^ States have implemented varied approaches to prevent prescription opioid misuse, including mandatory query of prescription drug monitoring programs prior to issuing opioid analgesic prescriptions and establishing limits on the quantity and days’ supply of opioid analgesics.^3,4,5^ These prescription opioid supply-side interventions, however, are estimated to be associated with modest decreases in overdose deaths, as recent data show that the OOD crisis is now mainly driven by illicitly manufactured synthetics, such as fentanyl.^1^

A widespread public health response to the OOD crisis has focused on increasing naloxone access, generally through community-based naloxone programs in which naloxone kits were distributed by local agencies free of charge.^6^ Naloxone is a prescription drug that rapidly reverses the effects of opioids (both natural and synthetic) and is considered an antidote for OOD. Since 2010, states have made legal changes aimed at expanding access to naloxone and increasing its use by laypersons.^7,8^ For example, as of 2017, 49 states and the District of Columbia had enacted a naloxone access law authorizing pharmacists to dispense or distribute naloxone without a patient-specific prescription from another medical professional.^7^ However, recent data show that, despite these legal interventions, overall naloxone dispensing remains low. For example, using data from Symphony Health’s Pharmaceutical Audit Suite Prescription Monthly data, Xu et al^9^ showed that, in 2016, a total of 147 457 naloxone prescriptions were dispensed from retail pharmacies, which is approximately 46 per 100 000. Similarly, Freeman et al,^10^ using data from IQVIA’s national prescription audit, reported widely varying rates of naloxone dispensing between states, ranging from 2.2 per 100 000 in Hawaii to 244 per 100 000 in Virginia during the second quarter of 2017. In April 2018, the Office of the Surgeon General issued a public health advisory on naloxone and opioid overdose noting the limited availability of naloxone in communities and encouraged health care practitioners to play more active roles in increasing the awareness, possession, and use of naloxone.^11,12^

An additional public health intervention recommended by several US federal agencies that is being considered by many states is emphasizing coprescribing or codispensing of naloxone to patients at risk for OOD. In 2016, the Centers for Disease Control and Prevention published an opioid prescribing guideline that recommends prescribers consider a naloxone coprescription if a patient’s opioid dosage exceeds 50 morphine milligram equivalents per day.^13^ The Substance Abuse and Mental Health Services Administration also recommended naloxone coprescribing for individuals with a history of OOD or substance use disorder, those who are taking benzodiazepines with opioids, and those who are at risk for using a high-dose opioid when they are no longer tolerant (eg, patients leaving detoxification facilities, jails).^14^ In 2017, Virginia and Vermont became the first 2 states to mandate naloxone coprescription to opioid-receiving patients who have risk factors of OOD.^15,16^ Our objective for this study was to assess the association between these legally mandated interventions requiring naloxone coprescription and naloxone dispensing over time.

## Methods

This was a population-based, state-level observational cohort study. We assessed the association between the number of naloxone prescriptions dispensed and the legal requirements for naloxone coprescription using longitudinal data analysis. The unit of observation was state-month. This study was deemed not human subjects research by the University of Kentucky Institutional Review Board and therefore was exempt from institutional review board review and approval. This study followed the Strengthening the Reporting of Observational Studies in Epidemiology (STROBE) reporting guideline.

### Outcome

The outcome variable was the number of naloxone prescriptions dispensed. The data came from the IQVIA national prescription audit and included nationwide all-payer naloxone prescription transactions in retail pharmacies per month per state from January 1, 2011, to December 31, 2017.^17^ The national prescription audit reflects nearly 90% of all retail pharmacies in the United States, and the data are weighted to approximately 100% of all dispensing using the IQVIA weighting methods.^17^ The data set included information on payment type (eg, third-party coverage or self-pay).

### Exposure

The primary exposure of the study was the legal requirements for naloxone coprescription. During 2011-2017, 2 states implemented legal requirements affecting naloxone coprescribing practice. Virginia was the first state to mandate naloxone prescription for patients with 1 or more risk factors of OOD, including prior OOD, substance misuse, doses exceeding 120 morphine milligram equivalents per day, and concomitant benzodiazepine use (regulation effective date March 15, 2017).^10^ Subsequently, effective July 2017, Vermont required naloxone prescriptions for patients receiving opioid doses exceeding 90 morphine milligram equivalents per day and those receiving opioid and benzodiazepine prescriptions concurrently.^11^ For analysis, an indicator variable was created in which Virginia and Vermont were categorized as having the legal requirements for naloxone coprescription from the first calendar month that those requirements were effective (March and July 2017, respectively).

### Covariates

#### Naloxone Access Laws

Between 2011 and 2017, a number of states adopted laws aimed at increasing the availability of naloxone to laypersons.^7^ One of the most common legal interventions instituted authorizes pharmacists to dispense naloxone without a patient-specific prescription from a physician or other prescriber, using various mechanisms, such as a standing order or state-wide protocol. Third-party prescribing laws were also increasingly adopted by which a prescriber can issue a naloxone prescription to family members, friends, or caregivers of an individual at risk for OOD, who are most likely to be first responders in the event of an OOD. The data on effective dates of standing orders and third-party prescribing laws were obtained from the Prescription Drug Abuse Policy System website.^7^ In most states, the effective dates of a naloxone standing order and third-party prescribing law were the same or only a few months apart; therefore, we created a composite indicator variable to reflect the first calendar month that either the standing order or third-party prescribing law was effective.

#### Retail Prescription Opioid Distribution

To adjust for underlying variations in prescription opioid analgesic use between states, we included the total distributed dose of prescription opioid analgesics in the analysis. Because drugs in the opioid class have relatively different potencies, opioid analgesic doses were standardized by morphine milligram equivalent. The data were derived from the Automated Reports and Consolidated Ordering System (ARCOS), a program operated by the Drug Enforcement Administration. The ARCOS collects controlled substances transaction reports at the state level from drug manufacturers and distributors.^18^ For this study, total grams of prescription opioid analgesics (excluding buprenorphine) were extracted from the ARCOS before they were converted to morphine milligram equivalents.^19^ Buprenorphine was excluded because it is primarily used for medication-assisted treatment among patients with opioid use disorder.^20^

#### Opioid-Involved Overdose Deaths

It is likely that the rate of opioid-related overdose deaths is associated with naloxone dispensing, as well as state-level regulatory considerations, such as naloxone coprescription mandates. For this reason, state-specific crude death rates involving opioid-involved overdose for each year were adjusted in the analysis. Mortality data for US residents for 2011-2017 were obtained from the National Center for Health Statistics Multiple Cause of Death within the Centers for Disease Control and Prevention’s Wide-Ranging Online Data for Epidemiologic Research Online Database,^21^ which is based on information from death certificates filed in 50 states and the District of Columbia. Drug overdose deaths involving opioids were identified, in accordance with a previously published Centers for Disease Control and Prevention report,^22^ using the *International and Statistical Classification of Diseases, 10th Revision* (*ICD-10*) codes. First, deaths with drug overdose as the underlying cause were identified using *ICD-10* codes of X40 to X44 (unintentional), X60 to X64 (suicide), X85 (homicide), and Y10 to Y14 (undetermined intent). Of those codes, opioid-related deaths were identified based on *ICD-10* codes of T40.0 to T40.4 and T40.6 (opioids), including those for heroin (T40.1), prescription opioids (T40.2-40.3), and synthetic opioids, excluding methadone (T40.4).

#### Percentage of Naloxone Prescriptions Paid for by Third-Party Payers

Even if naloxone is coprescribed with opioids, the patient may choose not to fill the naloxone prescription if it is not covered by a third-party payer. The list prices of naloxone increased over time during the study observation period.^23^ As the prices increase, having third-party coverage for a naloxone prescription becomes essential for ensuring financial access to the medication. As such, naloxone dispensing is likely correlated with an individual’s access to coverage for the naloxone prescription. Therefore, we adjusted for the percentage of naloxone prescriptions paid by third-party payers, which was obtained from the IQVIA national prescription audit. Third-party payers included Medicare, Medicaid, and commercial health insurance programs.

### Statistical Analysis

Monthly unadjusted rates of naloxone dispensing per 100 000 standard population were estimated. We compared the trend between 4 groups: (1) Virginia, (2) Vermont, (3) the top 10 states (including the District of Columbia) with OOD deaths in 2016 (West Virginia, New Hampshire, Ohio, District of Columbia, Massachusetts, Maryland, Rhode Island, Maine, Connecticut, and Kentucky),^21^ and (4) all 39 remaining states. We used 2016 estimates to identify the top 10 states (including the District of Columbia) with OOD deaths to compare baseline differences prior to legal requirements for naloxone coprescription in 2017.

We used a population-averaged approach to investigate the association between legal requirements for naloxone coprescription and dispensing.^24^ Based on the observed overdispersion in the outcome variable (ie, variance greater than the mean), we calculated a generalized estimating equation (GEE) using the log-link function and negative binomial distribution.^25^ We assumed that the strength of correlation between consecutive measurements within a state would decrease as measurements become farther apart. For this reason, we specified the working covariance structure as the first-order autoregressive process. Robust SEs were clustered at the state level. In addition to the covariates described above, census region (Northeast, Midwest, South, and West) and time, expressed as the number of months from the beginning of the observation period, were adjusted in the regression analysis. The natural logarithm of state population was included as an offset term. Incidence rate ratios (IRRs) with associated 95% CIs and the mean marginal effects were estimated.

To test the robustness of the GEE model results, we used a negative binomial regression with fixed-effects model to estimate the association between legal requirements for naloxone coprescription and dispensing. Detailed model specification and results are available in the eMethods in the Supplement. The results of the fixed-effects model were consistent with the GEE model results. Statistical significance was set at 2-tailed *P* < .05. All analyses were performed using Stata/SE, version 13 (StataCorp).^26^

## Results

In a descriptive, unadjusted analysis, the rate of naloxone dispensing per 100 000 increased significantly on the implementation of legal requirements for naloxone coprescription (Figure). An estimated 88 naloxone prescriptions per 100 000 were dispensed in Virginia and 111 prescriptions per 100 000 were dispensed in Vermont during the first full month that the legal requirement was effective (April and July 2017, respectively). Meanwhile, in July 2017, 16 naloxone prescriptions per 100 000 were dispensed in the top 10 states (including the District of Columbia) with OOD deaths and 6 prescriptions per 100 000 in the remaining states—all without mandated naloxone prescribing. By December 2017, the dispensing rate per 100 000 decreased to 34 in Virginia and 25 in Vermont.

**Figure.**
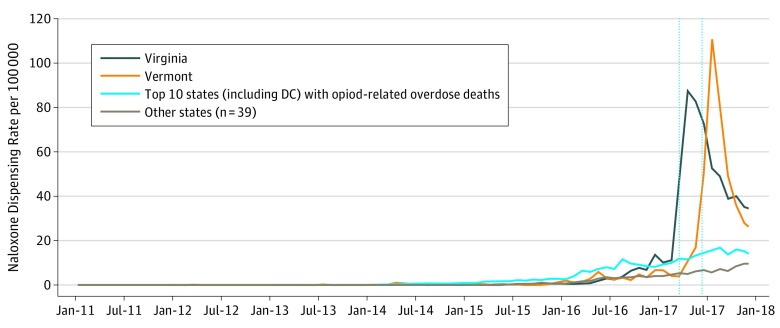
Naloxone Prescription Dispensing Rates per 100 000 Standard Population, 2011-2017 Legal requirements for naloxone coprescription became effective on March 15 and July 1, 2017, in Virginia and Vermont, respectively (shown as vertical lines on graph). The top 10 states (including the District of Columbia [DC]) with opioid-related overdose deaths include West Virginia, New Hampshire, Ohio, DC, Massachusetts, Maryland, Rhode Island, Maine, Connecticut, and Kentucky.

In the GEE regression model, the number of naloxone prescriptions dispensed was associated with the legal requirements for naloxone coprescription (IRR, 7.75; 95% CI, 1.22-49.35) (Table). In particular, implementation of the naloxone coprescription mandates was associated with an estimated 214 additional naloxone prescriptions dispensed per month in the period following the mandates, holding other variables constant. Among covariates, naloxone access laws (IRR, 1.37; 95% CI, 1.05-1.78), OOD death rates (IRR, 1.06; 95% CI, 1.04-1.08), the percentage of naloxone prescriptions paid by third-party payers (IRR, 1.009; 95% CI, 1.008-1.010), and time (IRR, 1.06; 95% CI, 1.05-1.07) were significantly associated with naloxone dispensing. The results of the GEE regression model were robust when compared with estimates from the negative binomial regression with the fixed-effects model (eTable in the Supplement).

**Table.** Population-Averaged Model for Variables Associated With the Number of Naloxone Prescriptions Dispensed, 2011-2017^a^

Variable	IRR (95% CI)	*P* Value
Legal mandate for naloxone coprescription	7.75 (1.22-49.35)	.03
Third-party prescribing or standing order law	1.37 (1.05-1.78)	.02
ln (total MME distributed)	0.92 (0.81-1.05)	.21
Crude death rate involving opioid overdose	1.06 (1.04-1.08)	<.001
% Naloxone paid by third-party payers	1.009 (1.008-1.010)	<.001
Time, mo	1.06 (1.05-1.07)	<.001
Region		
Northeast	1 [Reference]	
Midwest	0.97 (0.62-1.53)	.90
South	1.24 (0.77-2.00)	.38
West	1.48 (0.92-2.39)	.10
ln (population size)	1 (Offset term)	

Abbreviations: IRR, incidence rate ratio; MME, morphine milligram equivalent.

^a^ Generalized estimating equation used.

## Discussion

To mitigate the OOD crisis, states have implemented a variety of legal interventions aimed at increasing access to the opioid antagonist naloxone. Most recently, Virginia and Vermont mandated the coprescription of naloxone to potentially at-risk patients, including those with a history of OOD or receiving high-dose opioids. Our study suggests that legally mandating naloxone prescription for individuals at high risk for OOD is associated with an increased number of dispensed naloxone prescriptions. Our descriptive trend analysis suggests that the association is likely sequential, as the increase in the number of naloxone prescriptions dispensed was observed immediately on implementation of the legal mandates. In late 2018, similar mandates were enacted in California, Arizona, and Rhode Island.^27^ Further evaluation of the association between naloxone prescription and dispensing in these states with new mandates is now also warranted.

A decline in naloxone prescription dispensing was observed in the last quarter of 2017 in both Virginia and Vermont. This trend is anticipated to some extent, as the legal mandates for naloxone coprescription are at the patient level and not the prescription level.^15,16^ For example, the Vermont rule specifies that “prescribers shall coprescribe naloxone or document in the medical record that a patient has a valid prescription for or states they are in possession of naloxone.”^15^ Thus, it would be expected that once an initial naloxone coprescription was written and documented in the medical record, a prescriber would not issue a second naloxone prescription until such time the patient indicated that they no longer had a valid prescription or were no longer in possession of naloxone. Currently, data are limited as to when a second naloxone prescription is needed for persons meeting risk criteria. Our data set included the number of naloxone prescriptions dispensed in retail pharmacies, which is often less than the number of written prescriptions.^28,29,30^

In this study, having enacted either third-party prescribing or standing order laws was associated with a mean increase of 37% in naloxone dispensing. This result is in agreement with a recent study published by Xu et al.^9^ They conducted a longitudinal study using nationally representative pharmacy dispensing data and reported a 79% increase in naloxone dispensing as being associated with implementation of either third-party prescribing or standing orders for naloxone. While both studies report a positive association, the fact that the point estimate of the IRR reported by Xu et al^9^ is different from what we found is likely owing to differences in analytical approach (GEE vs fixed-effect), unit of observation (state-month vs state-quarter), and observation period (2011-2017 vs 2007-2016).

Particular caution is required when interpreting the IRR of the OOD deaths. The data on naloxone dispensing and OOD deaths are not temporal, and they do not suggest a sequence of those events. In other words, although naloxone dispensing increased during the observation period, the extent to which naloxone dispensing is influenced by OOD overdose deaths or vice versa was not estimated in this study. Instead, the reported IRR only reflects a positive association between the 2 variables as they occur simultaneously.

While OOD was not examined as an outcome variable in this study, a recently published study used a quasi-experimental design to demonstrate that harm reduction policy strategies, including naloxone access laws and overdose Good Samaritan laws, were associated with a significant reduction in OOD.^31^ In particular, states with naloxone access laws had an average of 15% lower incidence of OOD compared with states without those laws. Because these laws directly target naloxone availability, it is possible to infer that the process of naloxone prescribing and dispensing serves as an intermediate step for reducing the rate of OOD. We further argue that legal requirements for naloxone coprescription may have a potentially greater influence on preventing OOD through increased naloxone access for at-risk individuals.

### Limitations

There are limitations to this study. First, the strength of association reported in this study reflects the short-term assessment of the legal requirements for naloxone coprescription (<10 months). Further investigation is needed to evaluate the longer-term association. Second, our naloxone dispensing data do not include naloxone distributed free of charge through community programs. It is possible that the distribution of naloxone through such resources varies greatly by state, and as a result, states distributing a large quantity of naloxone for free appear as dispensing smaller numbers of naloxone prescriptions. The findings of this study should be applied only to naloxone dispensing in retail pharmacies. Third, the IQVIA data only report state-level counts of naloxone prescriptions. These data cannot be used to determine whether naloxone prescriptions were in fact coprescribed with opioids, since there are no individual-level patient identifiers that can be used to link naloxone and opioid prescriptions. In addition, these data lack diagnosis codes, such as opioid use disorder, that would allow determination of whether naloxone prescriptions are being filled by patients with illnesses at high risk for OOD. These limitations are substantial for the purposes of this analysis.

## Conclusions

Public policy efforts aimed at increasing naloxone access have focused on standing-order and third-party prescribing provisions. Although this study and others have shown these provisions to increase naloxone access,^9^ additional efforts are needed if we are to meet the US Surgeon General’s goal of broadening the availability and use of naloxone to reduce OOD mortality.^11,12^ Our study findings suggest that legally mandated naloxone prescription for persons at risk for OOD may increase naloxone dispensing and further reduce harm and save lives.

## Notes

Supplement. **eMethods.** Further Estimations **eTable.** Negative Binomial Regression With Fixed Effects Model to Estimate the Number of Naloxone Prescriptions Dispensed
