# Supplementary material for: Association of Naloxone Coprescription Laws With Naloxone Prescription Dispensing in the United States
Source: JAMA Netw Open. 2019 Jun 21;2(6):e196215. doi: 10.1001/jamanetworkopen.2019.6215 (PMC6593960; doi:10.1001/jamanetworkopen.2019.6215)
Supplement: Supplement. — eMethods. Further Estimations eTable. Negative Binomial Regression With Fixed Effects Model to Estimate the Number of Naloxone Prescriptions Dispensed [file jamanetwopen-2-e196215-s001.pdf]

Supplementary Online Content

Sohn M, Talbert JC, Huang Z, Lofwall MR, Freeman PR. Association of naloxone coprescription laws with naloxone prescription dispensing in the United States. *JAMA Netw Open*. 2019;2(6):e196215. doi:10.1001/jamanetworkopen.2019.6215

eMethods. Further Estimations

eTable. Negative Binomial Regression With Fixed Effects Model to Estimate the Number of Naloxone Prescriptions Dispensed

This supplementary material has been provided by the authors to give readers additional information about their work.

© 2019 Sohn M et al. *JAMA Network Open.*

eMethods. Further Estimations

In addition to the generalized estimating equation (GEE) model, we estimated the association between legal mandates for naloxone co-prescription and naloxone dispensing using a negative binomial regression with fixed effects model (robustness check). More specifically, the regression model included state-fixed effects, time-fixed effects (e.g., calendar month-specific dummy variables), covariates described in Methods of the article, and the natural logarithm of state population as an offset term. Incidence rate ratios (IRR) with associated 95% confidence intervals were estimated. The regression results are shown in the eTable.

© 2019 Sohn M et al. *JAMA Network Open.*

eTable. Negative Binomial Regression With Fixed Effects Model to Estimate the Number of Naloxone Prescriptions Dispensed, 2011-2017[1]

**Dependent variable: number of naloxone dispensing**		
**Variable**	**IRR (95% CI)**	**P-value**
Legal mandate for naloxone co-prescription	6.98 (4.88 – 9.99)	<0.001
Third-party prescribing or standing order law	1.22 (1.08 – 1.38)	0.002
ln(Total MME distributed)	0.74 (0.51 – 1.09)	0.132
Crude death rate involving opioid overdose	1.05 (1.04 – 1.07)	<0.001
% Naloxone paid by third-party payers	1.01 (1.01 – 1.02)	<0.001
Ln(Population size)	1 (offset term)	

Abbreviation: IRR, incidence rate ratio; 95% CI, 95% confidence interval; MME, morphine milligram equivalent.

[1]In addition to the variables in the table, state-fixed effects, and time-fixed effects were included in each model. Variables of Census region (Northeast, Midwest, South and West) were dropped because they do not vary within a state.

© 2019 Sohn M et al. *JAMA Network Open.*
